# The in- and outpatient health care use of patients with COPD before and after initiation of home care: a registry study from Norway

**DOI:** 10.1080/02813432.2024.2404056

**Published:** 2024-09-16

**Authors:** Tron Anders Moger, Jon Helgheim Holte, Olav Amundsen, Silje Bjørnsen Haavaag, Øystein Døhl, Line Kildal Bragstad, Ragnhild Hellesø, Nina Køpke Vøllestad, Trond Tjerbo

**Affiliations:** a Department of Health Management and Health Economics, Institute of Health and Society, University of Oslo, Oslo, Norway; b Department for Interdisciplinary Health Sciences, Institute of Health and Society, University of Oslo, Oslo, Norway; c Department of Public Health Science, Institute of Health and Society, University of Oslo, Oslo, Norway; d Department of Finance, City of Trondheim, Trondheim, Norway; e Department of Neuromedicine and Movement Science, Faculty of Medicine, Norwegian University of Science and Technology, Trondheim, Norway; f Department of Rehabilitation Science and Health Technology, Oslo Metropolitan University, Oslo, Norway

**Keywords:** COPD, registry data, outpatient care, home care, long-term care

## Abstract

**Objective:**

Chronic obstructive pulmonary disease (COPD) is a common condition associated with age, multimorbidity and frequently involves the use of health care across levels. Understanding the factors associated with the initiation of long-term care is important when planning the future need for services. We describe healthcare use before and after the reception of any home care. We further studied the associations between healthcare use and first registered home care service and from first registered home care service to nursing home admission or death.

**Design and subjects:**

Patients residing in Oslo or Trondheim at the time of first contact with a COPD primary diagnosis, 2009–2018. Patient data were linked across national and municipal registries, covering healthcare and sociodemographics. The sample consisted of 16,738 individuals.

**Results:**

There was a marked increase in inpatient and outpatient hospital contacts in the years prior to and after the reception of any home care. Adjusted for comorbidities and sociodemographics, high numbers of GP consultations, and inpatient and outpatient hospital contacts for respiratory diagnoses were associated with a significantly higher likelihood of receiving home care the next year (hazard odds ratios > 1.3). Following the reception of home care, the type of home care service received (e.g. home nursing or short-term rehabilitation/treatment) was more important than outpatient services in predicting next-year nursing home admission or death.

**Conclusion:**

Including data on prior outpatient care when predicting future need for home care is beneficial. A high frequency (top 10%) of yearly GP, in- or outpatient hospital contacts can imply that the patient may be in need of home care in the near future.

## Introduction

Chronic obstructive pulmonary disease (COPD) is a heterogeneous lung condition characterized by chronic respiratory symptoms due to abnormalities in the airway and/or bronchioles that cause persistent, often progressive, airflow obstruction [1]. Tobacco smoking is the most important risk factor in high-income countries. Other risk factors include biomass exposure, occupational exposures, air pollution and genetic factors [1]. COPD is currently the third leading cause of death worldwide, with more than 3 million deaths and 390 million cases [2,3]. In Norway, COPD is estimated to affect 6-7% of the population over 40 years of age, and more than 200,000 live with the disease [4]. These high rates cause major economic and societal burden [5,6]. In 2011, total direct healthcare costs were estimated at about €23.3 billion in the EU, with outpatient costs being slightly higher than drug and inpatient costs. The total amounted to an average yearly cost per patient of approximately €1000 [7]. In the US, the total spending on COPD in 2016 was estimated to be $34.3 billion [8] and around €275 million in Norway in 2019 [9]. The burden is expected to increase in the near future due to aging of the population as the prevalence increases with age, and more individuals are living with the long-term effects of COPD [1,5].

In most Western countries, there is an increased focus on the challenges and costs of providing sufficient long-term care (both home-based and institutional) in the face of ageing populations [10–15]. There is also a shift towards care that enables patients to live at home, both with and without home care. Norway saw a 40% increase in expenditure on home care from to 2015-2022, adjusted for inflation [16]. Increasing nursing home capacity at the same rate as the increase in the elderly in need of formal care is unfeasible. Identifying future care recipients when the need for long-term care arises requires good communication and coordination between family caregivers and primary and long-term care providers [17,18], something that is challenging because of inadequate channels for sharing information between levels of health care. Further complexity is added to the multimorbidity of recipients [12,18]. Multimorbidity, including cardiovascular disease, osteoporosis, depression, anxiety, and lung cancer, is frequent in COPD patients [1].

Research on factors associated with a higher risk of receiving home care in the general population has mainly focused on sociodemographic and economic factors, informal care from spouses and families, needs, functioning, and comorbidities [19–25]. To the best of our knowledge, the association between reception of home care and prior outpatient health service use has not been studied in population-based samples, neither for COPD patients nor in general. Moreover, data on prior outpatient health service use have rarely been included in the more extensive literature on risk factors for institutional care (nursing home) and hospitalization. This may have hampered previous research by predicting the future risk of health service use more accurately. Lin et al. (2022), for example, highlighted that predictive models for hospitalization and emergency department visits should be built on outpatient data, of which primary care is regarded as the most robust and applicable [26].

Recently, based on insights from research on the risk of future health service use enabled by the rapid development of information technology, efforts have been made to develop dashboard-type models that enable healthcare professionals to access important patient statistics in real time. This may assist healthcare professionals in the early identification of patients who are likely to benefit from different types of health and care services [27]. By facilitating early interventions at the lowest and most affordable care level, such models may help prevent or postpone more extensive use of healthcare services later in life. This may be beneficial for patients in terms of improved health outcomes and society in the form of lower costs. However, it is important to understand the extent to which the characteristics highlighted in these models actually predict future health service utilization and whether there are ways to enhance the information on these dashboards [27].

In Norway, it is possible to link health register data at an individual level using the national id-number and construct event histories for primary, specialist, and long-term care back to 2008. Hence, one may capture the use of in- and outpatient care several years prior to the reception of home care. Given the gaps in the literature outlined above, the aim of the present paper is to identify and describe factors in the in- and outpatient health service use, in terms of frequency and type of provider, associated with the likelihood of receiving home care during the following year. If these factors are strong predictors even after adjusting for comorbidities and sociodemographic and economic variables, it would be important to include health service use in models when planning the future need for home care in the municipality. This could also have implications for the individual patient. If an increase in contact frequency by certain types of in- and outpatient providers predicts that the patient may need home care in the near future, it will be relevant information for the service providers in the municipality in order to plan and organise their services more efficiently. This is particularly relevant for COPD, a common disease that should be followed up and treated in outpatient care [28]. Given that home care has been provided, we also studied associations between home care, in- and outpatient services, and next-year admission/death without admission to nursing homes in a second analysis. This will provide additional insight into whether the same healthcare factors are important predictors of the future health status for the patient also after home care has been initiated. Also, it will indicate within which type of services particularly vulnerable patients can be identified in administrative data.

## Material and methods

### The Norwegian setting

In Norway, primary and long-term care is the responsibility of municipalities and is largely funded by the central state through the municipal income system. The goal of this system is to level out inequalities based on demand and income in the municipalities and make all municipalities equally able to provide healthcare. Municipalities are required by law to provide necessary primary and long-term care services to their citizens. Home care covers health and medical services as well as general assistance to be able to live at home as the functional level declines. Services are either free or financed through limited income-dependent co-payments. All residents can apply for home services without referral from health providers. However, municipalities have the authority to define the need, amount, and type of care (both home and institutional).

All citizens are entitled to enlistment with a regular GP. Most GPs are independent contractors, not municipal employees. The financing of independent GPs is based on a combination of capitation and fee-for-service. The GP acts as a gatekeeper to other outpatient services, such as contract specialists, physiotherapists (referral from GP mandatory until 2019), and outpatient contacts with hospitals. Contract specialists are independent specialists on contract with a regional health authority. The number of fully private specialists is limited in Norway and not available in the data. There is patient co-payment for the services, with a total ceiling of roughly 300 EUR per year. Inpatient care is free of charge for the patient. Municipalities also operate primary care emergency rooms, which can be contacted without appointments or after hours.

Both the provision and financing of specialist care are conducted by the central state. Four regional health authorities were responsible for providing specialist care to the inhabitants of their region. These regional health authorities are financed through a combination of a block grant system and financing based on diagnosis-related groups (40% by 2023).

### Data

Patients residing in Oslo or Trondheim at the time of first contact with a COPD primary diagnosis (ICD-10: J43, J44, ICPC-2: R95) between 2009-2018 were identified from the Norwegian Patient Registry (NPR, in- and outpatient treatment, private rehabilitation) and the Control and Payment of Reimbursement to Health Service Providers (KUHR, contacts with GPs, contract specialists, and physiotherapists). In addition, patients could not receive any home care at inclusion and should have at least one year of follow-up from the first health contact with a COPD diagnosis. The individuals in the sample were followed through 2019. The sample was restricted to individuals between 40 years of age and censored at 95 years of age.

We acquired data on long-term care services from municipality electronic patient journals (MEPJ) in Oslo and Trondheim. All municipalities were required to register and report these data routinely to the Directorate of Health. However, to link long-term care data by national id number to other registers prior to 2018, one needs approval and access directly from the municipalities. We were able to access the data by collaborating with the departments of health in Oslo and Trondheim. Oslo is the most populous municipality in Norway, whereas Trondheim is the third most populous municipality in Norway. Sociodemographic and economic data were acquired from Statistics Norway (SSB) as well as the time of death from the Cause of Death Registry.

### Dependent variable

The outcome in the first analysis was the reception of any type of home care service (yes/no) from the municipality, ranging from safety alarms to short-term institutionalized care (e.g. rehabilitation stays, day center) for patients living at home. In the second analysis, the outcome was admission to a nursing home or death (yes or no), whichever occurred first. Both are absorbing states, precluding later reception of home services.

### Independent variables

To distinguish between COPD-related care and other care when describing the frequency and types of providers involved in treatment prior to the reception of home care, all inpatient and outpatient services were split into contacts with respiratory main diagnoses (ICD-10: J, ICPC-2: R) and non-respiratory diagnoses. In primary outpatient care, the provider must register a primary diagnosis to get reimbursed. Hence, most contacts in KUHR included only one diagnosis. For specialist care, the primary and the first of the secondary diagnoses were used to separate COPD-related care from other care. Consultations with GPs were identified using fee codes in KUHR. Other types of outpatient care include contact with contract specialists, physiotherapy, emergency rooms operated by the municipality (all in KUHR), outpatient contacts with the hospital, and rehabilitation (in NPR). Rehabilitation includes outpatient and inpatient rehabilitation in hospitals and rehabilitation in private clinics, in contract with the regional health authority. The rehabilitation content is unknown. Similarly, outpatient contacts with hospitals excluded those where the primary focus was rehabilitation (ICD-10 code Z50). Hospital admissions were counted as one if the discharge and admission dates were less than a day apart.

The independent variables and their definitions are summarized in the Supplemental Table S1. In addition to variables on healthcare frequency, we defined indicators for the type of home care service provided and for assistance in daily living based on the activities of daily living (ADL) checklist used by the municipality. The latter was only included in the analysis of associations with nursing home/death, as it is scored when home care services are provided. We also included sociodemographic and economic variables such as sex, income, education, and marital status from Statistics Norway, and a list of comorbidities commonly associated with COPD [29] based on ICD-10 and ICPC-2 codes in NPR and KUHR (Table S2).

### Statistical methods

Descriptive statistics for the sample are presented as means and standard deviations for continuous variables, and as percentages for categorical variables. We compared the characteristics of patients receiving home care during follow-up to those who did not receive home care and presented the most common services initially received from the municipality.

To describe the trend in the frequency of services used prior to the reception of home care (regardless of diagnosis registered for the contact), the mean number of contacts with different types of services in the five years before and after the date of the first registered home care service is presented. The first and third quartiles are reported to indicate the variation and skewness in service use. Patients entering the sample for less than five years prior to receiving services contribute to the estimates for the complete years where data are available. We assume that the 10-year data period is arbitrary, and that the representativeness of the sample is not affected by the number of years each person is under observation. For comparison, we also present the corresponding trend for a control sample of patients not yet receiving home care. This was based on observations with the same age distribution as those receiving home care each preceding year *i* = 1-5. Each observation for a patient receiving home care within *i* years was randomly matched without replacement by age to an observation for a patient not receiving home care until at least *i* + 1 years later. Hence, observations from patients who received home care later, either within or outside the follow-up period, may be included in the control sample.

In the analysis of associations between the independent variables and next year reception of any home care service, discrete time logit models were applied. The data were organized per year of follow-up and time-dependent variables were updated yearly in the models to capture changes over time. Indicator variables for each comorbidity were based on diagnoses in yearly healthcare contacts. The timescale was patient age, which was included using dummy variables for each year. Censoring occurred at the end of follow-up or death. As the aim was to identify risk factors associated with the reception of home care, death prior to receiving services was not analyzed as a competing risk. These patients do not consume any long-term care resources.

Discrete time logit models were also applied in the analysis of associations between the independent variables and the second outcome (next-year admission to nursing home or death). However, the indicators for types of home care received and ADL scores were updated quarterly in the models to capture changes in service provision over time. This was done because several of these may be provided only for a short period of time.

In the logit models, the frequency of service use per year was further organized as follows: consultations with GP per year were categorized according to being above the 75th, 95th- and 99th-percentile. This was done because of the great variation in GP consultations, and thus, to study the effects of being in the tail of the distribution; the difference in likelihood of receiving services if seeing the GP once or twice in a year is presumably of little relevance. Other services are used much more rarely; hence, contacts with contract specialists, physiotherapists, outpatient contacts with hospitals, rehabilitation (private or in-hospital), and in-hospital admissions were categorized into more than one/one or no contact in a year. More than one contact in a year corresponds to being above the 75^th^-percentile for non-respiratory contract specialists and outpatient hospital contacts and above the 90^th^-percentile for the other variables. To illustrate the predictive value of the variables describing health service use, area under the curve (AUC), negative (NPV), and positive (PPV) predictive values were compared among five models: the full model with all independent variables, a model with all comorbidities and sociodemographic and economic variables, a model with all health service variables and age dummies, a model with the two types of GP consultations and age dummies, and a model with age dummies only. NPV and PPV were calculated at the probability cut-off in the AUC where the sensitivity and specificity were approximately equal (and hence assumed to be equally important).

The data were analyzed using Stata version 16.1, and a 5% significance level was used throughout.

## Results

The descriptive statistics for the raw samples are presented in Table 1. In total, there were 16,738 individuals, 11,447 who did not receive home care and 5,291 individuals where home care was initiated during follow-up. Not surprisingly, the age at first contact with COPD was substantially higher among patients receiving home care. Income and education levels were lower, while the proportion of widows/widowers was higher. The proportion of patients on permanent disability pensions was also markedly higher among those receiving home care. and there was a tendency of higher use of most health services. However, there was little difference in the number of comorbidities between the groups. After the initiation of home care, there was a marked increase in the number of comorbidities (see Table S2 for the prevalences), emergency room contacts, hospital admissions and outpatient contacts with hospitals for non-respiratory diagnoses. Only 12% were admitted to a nursing home. The most frequent services initially received by the patients are shown in Table S3.

**Table 1.** Descriptive statistics for COPD patients with no registered home care during follow-up and for patients where home care was initiated.

	No home care (*n* = 11,447)	Home care (*n* = 5,291)	
		Before initiation	After initiation
Variable:	Mean/% (SD)	Mean/% (SD)	Mean/% (SD)
Years of follow-up	6.3 (3.1)	3.9 (2.5)	3.3 (2.6)
	**Sociodemography and -economy**		
Age at first COPD diagnosis in data	60.8 (9.9)	69.1 (9.6)	–
Male	50%	47%	–
Income	555k	356k	–
Education: Primary	31%	39%	–
Secondary	45%	45%	–
University/college	24%	16%	–
Married/cohabitant	48%	44%	–
Widow/widower	5%	17%	–
Not married/divorced	47%	39%	–
Permanent disability pension	35%	35%	–
Dead prior to home service/nursing home	7%	–	39%
Admitted to nursing home	–	–	12%
Sum of comorbidities	2.4 (1.4)	2.5 (1.5)	3.3 (1.8)
	**Health care use**		
GP consultations, respiratory diagnoses	1.5 (1.5)	2.0 (2.3)	2.0 (3.1)
GP consultations, non-respiratory diagnoses	3.8 (3.6)	4.9 (4.8)	5.3 (5.7)
Municipal emergency room contacts, respiratory diagnoses	0.03 (0.1)	0.06 (0.3)	0.3 (1.3)
Municipal emergency room contacts, non-respiratory diagnoses	0.3 (0.8)	0.3 (0.9)	2.0 (7.6)
Physiotherapy contacts, respiratory diagnoses	1.2 (6.3)	1.9 (8.1)	1.5 (7.7)
Physiotherapy contacts, non-respiratory diagnoses	3.7 (9.7)	3.3 (10.0)	3.4 (11.3)
Contract specialist contacts, respiratory diagnoses	0.3 (0.7)	0.2 (0.7)	0.2 (0.7)
Contract specialist contacts, non-respiratory diagnoses	1.3 (2.5)	1.5 (2.7)	1.3 (2.6)
Outpatient hospital contacts, respiratory diagnoses	0.3 (1.2)	0.5 (1.8)	0.8 (2.9)
Outpatient hospital contacts, non-respiratory diagnoses	2.1 (3.6)	2.6 (4.6)	6.6 (11.6)
Rehabilitation, respiratory diagnoses	0.06 (0.4)	0.1 (0.5)	0.1 (0.7)
Rehabilitation, non-respiratory diagnoses	0.1 (0.8)	0.1 (0.8)	0.2 (1.2)
Hospital admissions, COPD	0.05 (0.2)	0.2 (0.5)	0.7 (1.9)
Hospital admissions, respiratory diagnoses	0.1 (0.3)	0.3 (0.6)	1.3 (2.8)
Hospital admissions, non-respiratory diagnoses	0.3 (0.6)	0.4 (2.2)	1.7 (11.1)

For the latter group, statistics are shown during the period before and after home care was initiated. Health care contacts are averages per follow-up year. SD = standard deviation.

**Figure 1.**
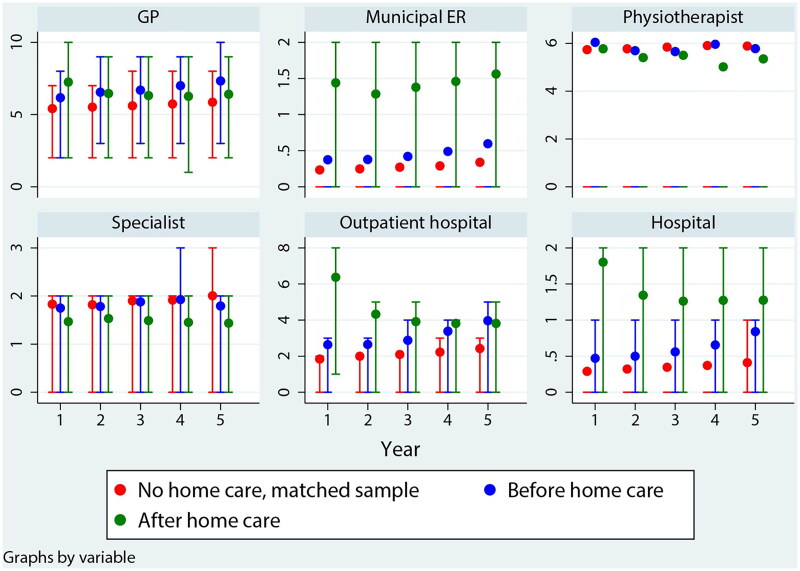
Mean (dot), 1^st^ and 3^rd^ quartile (bars) number of contacts for different types of services, 5 years prior to the initiation of home care (blue) and five years after (green). For comparison, no home care, matched sample (red) are observations with the same age distribution as those receiving home care during the next 1–5 years, but where the reception of services is at least 2–6 years into the future.

Figure 1 shows the trend in healthcare use (respiratory and non-respiratory diagnoses in total) for the five years prior to home care, compared with the two control samples. There was a trend towards increasing use of GP contacts, emergency room contacts, outpatient hospital contacts, and hospital admissions when approaching the start of home care compared to the sample not receiving home care. For physiotherapy and contract specialist contact, there was no clear trend. Use of emergency room and physiotherapy was particularly skewed, with the 3^rd^ quartile at zero.

Table 2 shows the results for the variables on health service frequency per year (for reference, the unadjusted results are given in Table S4). Most variables, regardless of being with a respiratory or other diagnosis, were associated with significantly increased odds of receiving home care adjusted for comorbidities and sociodemographic and economic factors. However, contract specialists, diagnoses, physiotherapy contacts, and non-respiratory diagnoses were associated with a decrease in the odds of receiving home care. For the outcome of nursing home admission or death, increasing numbers of GP consultations, emergency room contacts (respiratory diagnoses), and hospital admissions (any diagnosis) were associated with increased odds. In addition, emergency room contacts, contract specialists, and physiotherapy contacts with non-respiratory diagnoses were associated with decreased odds of outcome.

**Table 2.** Regression results for health service variables, adjusted for variables in Table 3 (nursing home or death) and Table S5.

Outcome:	Home care (*n* = 90,081 observations from 16,738 individuals)	Nursing home or death (*n* = 66,129 observations from 5,291 individuals)
Health service:	Odds ratio (95%-CI)	
GP, respiratory diagnoses		
3-5 vs. 0-2	1.10 (1.02-1.16)*	1.10 (0.97-1.24)
6-10 GP vs. 0-2	1.39 (1.24-1.55)*	1.24 (1.02-1.41)*
11+ GP vs. 0-2	1.42 (1.13-1.80)*	1.38 (0.95-1.63)*
GP, non-respiratory diagnoses		
6-11 GP vs. 0-5	1.20 (1.11-1.30)*	0.90 (0.81-1.02)
12-19 GP vs. 0-5	1.56 (1.39-1.75)*	0.97 (0.81-1.12)
20+ GP vs. 0-5	1.62 (1.32-1.97)*	0.98 (0.81-1.35)
Municipal emergency room, respiratory	1.35 (1.16-1.59)*	1.23 (1.06-1.44)*
Municipal emergency room, non-respiratory	0.95 (0.88-1.03)	0.55 (0.49-0.61)*
Contract specialist, respiratory	0.90 (0.83-0.99)*	1.19 (0.99-1.42)
Contract specialist, non-respiratory	0.83 (0.78-0.89)*	0.79 (0.72-0.87)*
Physiotherapist, respiratory	1.20 (1.07-1.34)*	0.85 (0.70-1.03)
Physiotherapist, non-respiratory	0.87 (0.81-0.95)*	0.71 (0.61-0.83)*
Outpatient hospital, respiratory	1.34 (1.24-1.45)*	1.08 (0.97-1.20)
Outpatient hospital, non-respiratory	1.14 (1.06-1.22)*	0.92 (0.82-1.04)
Hospital admissions, respiratory	1.52 (1.40-1.66)*	1.67 (1.50-1.86)*
Hospital admissions, non-respiratory	1.21 (1.12-1.31)*	1.36 (1.23-1.51)*
Rehabilitation, respiratory	1.21 (1.00-1.47)*	0.96 (0.76-1.22)
Rehabilitation, non-respiratory	0.99 (0.82-1.19)	0.98 (0.94-1.01)

CI = Confidence interval. *Significant at 5%-level.

Table 3 shows associations of home care service variables to the outcome nursing home or death (unadjusted results are given in Table S4). Generally, these were associated with an increased odds of the outcome. In particular, the estimated hazard odds ratios for assisted living, respite care, home nursing and rehabilitation were much higher than for the outpatient service variables in Table 2. Results for the comorbidities, sociodemographic and economic variables are given Tables S5 and S6 in the Supplemental Material. Most comorbidities were associated with an increased odds, as expected.

**Table 3.** Regression results for home care service variables, adjusted for variables in Tables 2 and S5.

	Prevalence in sample (cumulative during follow-up from time of first service)	Nursing home or death (*n* = 66,129 observations from 5,291 individuals)
**Home care service**:		Odds ratio (95%-CI)
Safety alarm	48%	1.00 (0.90-1.10)
Any assistance at home	44%	1.11 (1.01-1.23)*
Assisted living	3%	1.65 (1.31-2.07)*
Short-term day or night stay, institution	2%	1.31 (0.82-2.09)
Respite care in/out of institution	3%	2.65 (1.93-3.64)*
Home nursing	67%	2.88 (2.62-3.17)*
Short-term rehabilitation/treatment in/outside institution	53%	1.45 (1.40-1.50)*

CI = Confidence interval. *Significant at 5%-level.

Table 4 shows the AUC, NPV, and PPV to illustrate the predictive ability of different sets of variables in the models. The full models had AUC values of 0.79 for the outcome of receiving home care, and 0.82 for the outcome of nursing home or death, respectively. The reduction in AUC for models including comorbidities, sociodemographic and economic variables, or health service variables only was slight for both outcomes. The model including only GP consultations and age performed reasonably well compared with the larger models in predicting reception of home care, but less so for the outcome nursing home or death.

**Table 4.** Comparison of different models by area under the curve (AUC), negative predictive value (NPV) and positive predictive value (PPV).

Outcome:	Home care			Nursing home or death		
Model	AUC	NPV	PPV	AUC	NPV	PPV
Full model	0.79	72.2%	70.1%	0.82	76.2%	72.4%
Comorbidities and sociodemographic and economic variables	0.77	69.2%	70.1%	0.76	71.1%	68.5%
Health service variables and age dummies	0.76	69.4%	69.3%	0.79	76.3%	67.6%
GP consultations, respiratory and non-respiratory diagnoses and age dummies	0.73	67.1%	67.2%	0.59	57.4%	53.8%
Age dummies only	0.71	64.4%	67.1%	0.58	60.4%	50.4%

NPV and PPV calculated at the probability cut-off where sensitivity and specificity were approximately equal. For the outcome nursing home or death, the health service variables include home care.

## Discussion

### Main findings

Our results indicate that data on GP consultations, emergency rooms, outpatient hospital contacts and hospital admissions provide additional information relevant for identifying patients in need for home care, information not captured by comorbidities, and sociodemographic and economic variables. This suggest that data on health service use should be included in predictive models for COPD patients. The fact that several of the health service variables showed relatively large associations to the outcomes stresses the importance of having available data across outpatient, inpatient and home care providers to improve precision in predictions. This implies that the health providers in the municipality should not plan the home care for COPD patients without considering the relationship to the other services, as several providers interact in the follow-up for this patient group both prior to and after the initiation of home-based services. The results further suggest that variables on the yearly frequency of health service use, in crude categories, have a similar predictive ability as indicators of common comorbidities and sociodemographic and economic variables. As the healthcare service variables were categorized, the results imply that relatively many contacts (e.g. above the 90^th^ percentile) are required to substantially alter the likelihood of home care. Relatively few COPD patients will be flagged as high risk from the health service variables in the model. This should be an advantage if using data on healthcare services when planning the need for future home care, both in terms of volume, for identifying vulnerable patients in administrative data, and overall monitoring of the patient group in the municipality.

### Findings in relation to the literature

To our knowledge, this is the first study to examine the characteristics of outpatient care and its association with later reception of home care in a large, population-based sample, both for COPD patients but also more generally. A number of quantitative studies have examined factors associated with the level of care (e.g. for home care [30,31], use of home-based and out-of-home respite care [32]), progression of care (e.g. from home to institution [33], or from home care to institution [33]), or the effectiveness of particular interventions (e.g. home care by outreach nursing for COPD patients [34]). Some of these studies included prior hospitalization or types of home care among the factors studied; however, outpatient care was lacking. Hence, we believe that this study covers a blind spot in the literature.

The findings for comorbidities and sociodemographic and economic variables are in line with the literature [19–25]. Within the frequently used Andersen-Newman framework for identifying and structuring individual determinants of health service use [35,36], these factors cover predisposing characteristics (e.g. age and sex), enabling resources (e.g. marital status, informal care), and need (e.g. comorbidities, activities of daily living used by the home care provider to decide the level of services). In particular, the variables on the use of primary care (GPs, physiotherapists, and municipal emergency rooms) are, to some extent, both need and enabling factors. On the one hand, they could reflect needs due to poorer health status. COPD is a chronic disease, and many comorbidities are not only frequent in the multimorbidity of COPD patients but are also chronic conditions themselves. Hence, the associations with frequency of healthcare use for both respiratory and non-respiratory diagnoses in the patient group could be due to the cumulative burden of disease progression, thus requiring an increasing number of healthcare contacts, rather than just COPD and comorbidities being present in the patient. If this is the case, it implies that one obtains better assessment of the patient’s health status by including the frequency of outpatient healthcare contacts in predictive models, compared to using only comorbidity indicators (whether used individually or as an index). However, the use of primary care can also represent enabling factors. Assistance from primary care providers can be important in the process of applying for and facilitating home care from the municipality in addition to family support. This is perhaps particularly important for COPD patients, as the relatively high frequencies of lifestyle diseases, depression, and mental disorders [1] indicate that they are at a disadvantage.

### Other findings

There was a substantial increase in the frequency of hospital admissions, emergency rooms, and outpatient hospital contact after the initiation of home care. Also, the types of home care provided by the municipality, such as home nursing and short-term rehabilitation/treatment spells, showed much stronger associations than most outpatient services for the outcome nursing home or death. This could partly be due to an abrupt change in health status, as around 75% of the patients had one or more hospital admissions during the prior year (not shown). However, as hospital admissions, emergency room and outpatient hospital contacts were more frequently also in the following years, it may alternatively indicate that assessments are made differently when home care providers are involved. For instance, there could be a lower threshold for transferring the patient to specialist care.

The results further suggest that contract specialists (any diagnosis) and physiotherapy contacts (non-respiratory diagnoses) were associated with decreased odds of receiving home care. The same applies to emergency rooms, contract specialists, and physiotherapy contacts regarding the outcome of nursing homes or death. A possible explanation could be that patients using contract specialists or physiotherapists are healthier or more resourceful, and hence, their health status and socioeconomic factors are inadequately captured or modelled in the analysis. However, reconsidering the analysis only on observations where home nursing or rehabilitation was provided from the municipality (both should imply poor health at least temporarily) did not alter these effects. Regarding the finding of emergency room contacts, one could imagine confounding effects between service variables with and without respiratory diagnoses. Including health service variables only for non-respiratory diagnoses did not substantially alter the estimates. Rather, the results seem to suggest that respiratory healthcare for COPD patients is more important than healthcare related to comorbidities for both outcomes.

### Methodological considerations

The choice of organizing the data per year in the analysis was, to some extent, pragmatic. Exact dates were available for all contacts with different types of services. However, because of the number of time-dependent variables involved, performing a continuous-time survival analysis would be very challenging, and also with uncertain gains given that the primary focus was on associations with the frequency and type of provider. The discrete time logit model, rather than the more common complementary log-log model yielding discrete time proportional hazards, was used because the logit model indicated a slightly better fit based on AIC values. The independent variable estimates were similar in terms of both magnitude and significance. The follow-up period was relatively short given the aim of estimating associations with the use of home care. Whether severe non-proportionality by age was present in the models was difficult to assess with certainty, as the coefficients were estimated from patients observed for only 1-10 years and pieced together across the age range.

### Strengths and limitations

The main strength of the study is the extensive data and the fact that they are linked at the individual level. For most patients, the history of health care use is known several years in advance of the initiation of home care, and this information is utilized in the analyses. It also enables the analysis of a general sample of COPD patients, including both newly diagnosed and old cases of all ages. Even though we included patients within a wide age range, the regression results were similar if restricting the sample to patients aged 40-60 or above 80 years. Hence, the main conclusions seem applicable for the general sample.

The two main limitations of the study were the lack of clinical COPD severity information and smoking status. This is a common challenge in administrative data, and it would be of interest to assess the association between COPD severity and likelihood of receiving home care, while also being able to adjust for smoking status in the models. Another limitation is the use of a primary care diagnosis as an inclusion criterion when counting the number of contacts with each service. The validity of ICPC-2 diagnoses is uncertain, and as most contacts in primary care include one diagnosis, there could be some degree of arbitrariness regarding which diagnosis is registered in the data when several conditions are present in the patient [38]. However, as the follow-up period was extended over several years for most patients in the sample, the most severe comorbidities should be captured. The prevalences of the comorbidities were also as high or substantially higher in the data from GPs, physiotherapists and contract specialists, compared with the data from outpatient hospital and inpatient care (NPR). Hence, the prevalence of comorbidities in patients receiving home care does not seem to be severely affected by the higher frequency of hospital contacts. Some of the healthiest patients, who are rarely in contact with health services primarily because of COPD, may not be included, and a significant proportion of COPD cases may be undiagnosed in the population [39].

Regarding generalizability, using only two municipalities has advantages and disadvantages. Heterogeneity in access to both primary and specialist care is reduced; hence, the strength of the associations with service frequencies estimated in the analysis should not be influenced by these factors. On the other hand, as the focus of this paper is to identify associations rather than causality, it could similarly be argued that the lack of nationwide home care data is a disadvantage, as it is not possible to assess whether the associations are similar across different parts of the country. Generalizability in other countries may be limited by variations in home care systems [37].

## Conclusion

We found strong associations between the yearly frequency of GP, municipal emergency room, in- and outpatient hospital contacts, and later reception of home care for patients with COPD. These associations remained after adjusting for common comorbidities and sociodemographic and economic variables. After receiving home care, the type of home care received seemed to be more important for predicting admission to nursing homes or death without prior nursing home admissions. Monitoring and having access to the history of healthcare contacts and type of service is important both when planning and predicting future need for home care and to better identify vulnerable patients in administrative data when doing analyses of outcomes in long-term care.

## Supplementary Material

Supplemental Material
