# Supplementary material for: The in- and outpatient health care use of patients with COPD before and after initiation of home care: a registry study from Norway
Source: Scand J Prim Health Care. 2024 Sep 16;43(1):100–10. doi: 10.1080/02813432.2024.2404056 (PMC11834786; doi:10.1080/02813432.2024.2404056)
Supplement: Supplemental Material [file IPRI_A_2404056_SM7232.docx]

**Appendix tables**

Table S1. List of independent variables used in the analysis. NPR=Norwegian Patient Registry, KUHR=Control and Payment of Reimbursement to Health Service Providers, RGP=Regular General Practitioner registry, MEPJ=municipality electronic patient journal, SSB=Statistics Norway.

| Variable | Definition | Source |
| --- | --- | --- |
| **Frequency of health service contacts per year** | | |
| Consultations with GP, respiratory diagnoses | Number of consultations and categories in three groups 0-2, 3-5, 6-10, 11+ | KUHR |
| Consultations with GP, non-respiratory diagnoses | Number of consultations and categories in three groups 0-5, 6-11, 12-19, 20+ | KUHR |
| Emergency room contacts, respiratory diagnoses | Number of contacts with municipal emergency room and categories 0-1 or 2+ | KUHR |
| Emergency room contacts, non-respiratory diagnoses | Number of contacts with municipal emergency room and categories 0-1 or 2+ | KUHR |
| Contact with contract specialist, respiratory diagnoses | Number of contacts and categories 0-1 or 2+ | KUHR |
| Contact with contract specialist, non-respiratory diagnoses | Number of contacts and categories 0-1 or 2+ | KUHR |
| Contact with physiotherapist, respiratory diagnoses | Number of contacts with general physiotherapist, manual physiotherapist, occupational therapist and categories 0-1 or 2+ | KUHR |
| Contact with physiotherapist, non-respiratory diagnoses | Number of contacts with general physiotherapist, manual physiotherapist, occupational therapist and categories 0-1 or 2+ | KUHR |
| Outpatient contact with hospital, respiratory diagnoses | Number of contacts and categories 0-1 or 2+ | NPR |
| Outpatient contact with hospital, non-respiratory diagnoses | Number of contacts and categories 0-1 or 2+ | NPR |
| Rehabilitation in hospital or private clinics on public contract | Number of stays and categories 0-1 or 2+ | NPR |
| Inpatient hospital admissions, respiratory diagnoses | Number of admissions and categories 0-1 or 2+ | NPR |
| Inpatient hospital admissions, non-respiratory diagnoses | Number of admissions and categories 0-1 or 2+ | NPR |
| **Home care service indicators** | | |
| Safety alarm | Quarterly indicator of receiving service | MEPJ |
| Any assistance at home | Quarterly indicator of receiving service | MEPJ |
| Assisted living | Quarterly indicator of receiving service | MEPJ |
| Short-term day or night stay, institution | Quarterly indicator of receiving service | MEPJ |
| Respite care in/out of institution | Quarterly indicator of receiving service | MEPJ |
| Home nursing | Quarterly indicator of receiving service | MEPJ |
| Short-term rehabilitation/treatment in/outside institution | Quarterly indicator of receiving service | MEPJ |
| **Multimorbidity, sociodemographic and –economic variables** | | |
| Comorbidities | Indicators for each of 17 categories of comorbidities, see Table A1 for details | NPR and KUHR |
| Age | Age in years | SSB |
| Sex | Indicator of male sex | SSB |
| Income | Gross income per year | SSB |
| Education when entering sample | Categorized as primary, secondary and university/college | SSB |
| Marital status when entering sample | Categorized as married/cohabitant (reference), widow/widower, not married/divorced | SSB |
| Disability pension when entering sample | Indicator for permanent disability pension | SSB |
| Need-score for assistance in daily living | Sum score of items in the activities of daily living (ADL) checklist, assume score of 0 if not receiving any long-term care service, updated quarterly | MEPJ |

Table S2. Definitions of comorbidities by ICD-10 and ICPC-2 codes. Prevalences are cumulative across follow-up prior to reception of home care services, and from reception of services to end of follow-up.

| **Variable:** | **ICD-10:** | **ICPC-2:** | **Prevalence prior to home care services (n=16,738)** | **Prevalence after home care services (n=5,291)** |
| --- | --- | --- | --- | --- |
| Alcoholism | F10-F17 | P15-P16 | 7% | 10% |
| Anemia | D5*-D6* | N80-N82 | 6% | 19% |
| Cancer (except lung cancer) | All C except C3 | A79, B72-B74, D74-D77, F74, H75, K72, L71, N74, R84-R85, S77, T71, U75-U77, X75-X77, Y77-Y78 | 18% | 32% |
| Cardiovascular disease | I20, I25, I48, I70-79 | K74-K76, K78-80, K92 | 33% | 48% |
| Dementia | F00-F03, G30 | P70 | 1% | 10% |
| Depression and anxiety | F32-F34, F40-F41 | P74, P76 | 19% | 20% |
| Diabetes | E10-E14 | T89-T90 | 15% | 17% |
| Heart failure | I50 | K77 | 9% | 26% |
| Hypertension | I10-I15 | K86-K87 | 41% | 40% |
| Kidney disease or failure | N18-19 | U88, U99 | 6% | 14% |
| Lung cancer | C3* | R84-R85 | 4% | 12% |
| Mental disorders | F20-F31 | P72-P73, P75, P77, P79-P80, P82, P98-P99 | 5% | 5% |
| Myocardial infarction (incl. angina pectoris) | I20-I22 | K74-K75 | 13% | 16% |
| Obesity | E65-E66 | T82 | 4% | 3% |
| Osteoporosis | M81 | L95 | 10% | 16% |
| Stroke | I60-I64, G45 | K89-K91 | 8% | 16% |
| Underweight | E40-E46 | T05, T08, T11, T28, T91, T99 | 23% | 34% |

Table S3. Most common home care services initially provided to the patients (n=5,291). More than one service may be provided as first service to each patient.

| Type of service: | % (n) |
| --- | --- |
| Home nursing | 32% (1,888) |
| Safety alarm | 17% (980) |
| Assistance, daily activities | 14% (817) |
| Rehabilitation at home/out of institution | 11% (632) |
| Time-limited stay for treatment in institution | 6% (378) |
| Others | 20% (1,168) |

Table S4. Unadjusted regression results for health service variables. CI=Confidence interval. *=significant at 5%-level.

| **Outcome:** | Home care services (n=90,081 observations from 16,738 individuals) | Nursing home or death (n=66,129 observations from 5,291 individuals) |
| --- | --- | --- |
| **Health service**: | Odds ratio (95%-CI) | |
|  | | |
| GP, respiratory diagnoses |  |  |
| 3-5 vs. 0-2 | 1.29 (1.20-1.39)* | 1.21 (1.09-1.34)* |
| 6-10 vs. 0-2 | 1.93 (1.73-2.14)* | 1.46 (1.27-1.68)* |
| 11+ vs. 0-2 | 2.58 (2.07-3.20)* | 1.90 (1.51-2.38)* |
| GP, non-respiratory diagnoses |  |  |
| 6-11 vs. 0-5 | 1.39 (1.29-1.49)* | 0.79 (0.79-0.87)* |
| 12-19 vs. 0-5 | 1.99 (1.79-2.21)* | 0.89 (0.78-1.02) |
| 20+ vs. 0-5 | 2.34 (1.94-2.83)* | 0.84 (0.66-1.06) |
| Municipal emergency room, respiratory | 2.35 (2.06-2.69)* | 1.56 (1.39-1.76)* |
| Municipal emergency room, non-respiratory | 1.54 (1.43-1.65)* | 1.11 (1.03-1.21)* |
| Contract specialist, respiratory | 0.82 (0.75-0.90)* | 0.83 (0.71-0.98)* |
| Contract specialist, non-respiratory | 0.86 (0.81-0.91)* | 0.63 (0.58-0.68)* |
| Physiotherapist, respiratory | 1.46 (1.31-1.63)* | 0.90 (0.76-1.06) |
| Physiotherapist, non-respiratory | 0.90 (0.83-0.97)* | 0.54 (0.47-0.62)* |
| Outpatient hospital, respiratory | 1.89 (1.76-2.03)* | 1.54 (1.41-1.68)* |
| Outpatient hospital, non-respiratory | 1.52 (1.43-1.61)* | 1.49 (1.35-1.64)* |
| Hospital admissions, respiratory | 2.47 (2.29-2.65)* | 2.61 (2.40-2.83)* |
| Hospital admissions, non-respiratory | 1.64 (1.54-1.76)* | 1.98 (1.83-2.15)* |
| Rehabilitation, respiratory | 2.06 (1.72-1.46)* | 1.13 (0.92-1.39) |
| Rehabilitation, non-respiratory | 1.36 (1.14-1.62)* | 0.98 (0.95-1.02) |
| **Home care services**: |  |  |
| Safety alarm | - | 1.33 (1.22-1.45)* |
| Any assistance at home | - | 1.68 (1.55-1.83)* |
| Assisted living | - | 2.71 (2.21-3.33)* |
| Short-term day or night stay, institution | - | 2.09 (1.36-3.22)* |
| Respite care in/out of institution | - | 5.30 (3.98-7.04)* |
| Home nursing | - | 4.81 (4.41-5.24)* |
| Short-term rehabilitation/treatment in/out of institution | - | 1.69 (1.64-1.75)* |

Table S5. Regression results for sociodemographic, –economic factors and comorbidities, adjusted for variables in Tables 2 and 3 (nursing home or death). CI=Confidence interval. *=significant at 5%-level

| **Outcome:** | | Home care services (n=90,081 observations from 16,738 individuals) | Nursing home or death (n=66,129 observations from 5,291 individuals) |
| --- | --- | --- | --- |
| **Independent variable:** | | Odds ratio (95%-CI) | |
|  | **Sociodemography and -economy** | | |
| Female gender | | 1.04 (0.98-1.11) | 0.84 (0.76-0.93)* |
| Income per 100,000 NOK | | 0.86 (0.84-0.88)* | 0.98 (0.95-1.02) |
| Education: Secondary vs. primary | | 0.88 (0.83-0.95)* | 1.34 (1.00-1.81) |
| Education: University/college vs. primary | | 0.82 (0.74-0.90)* | 1.47 (1.07-2.03)* |
| Marital status: Widow/widower vs. married | | 1.81 (1.65-1.99)* | 0.99 (0.87-1.12) |
| Marital status: Not married/divorced vs. married | | 1.43 (1.34-1.53)* | 1.00 (0.88-1.13) |
| Permanent disability pension | | 1.33 (1.24-1.54)* | 0.91 (0.79-1.04) |
|  | **Comorbidities** | | |
| Alcoholism | | 2.11 (1.81-2.47)* | 0.91 (0.74-1.13) |
| Anemia | | 1.20 (1.00-1.45) | 1.44 (1.25-1.65)* |
| Cancer | | 1.15 (1.05-1.26)* | 1.90 (1.71-2.11)* |
| Cardiovascular disease | | 1.04 (0.96-1.13) | 1.00 (0.90-1.11) |
| Dementia | | 3.57 (2.66-4.78)* | 1.80 (1.55-2.09)* |
| Depression | | 1.54 (1.39-1.70)* | 0.86 (0.74-1.00) |
| Diabetes | | 1.27 (1.16-1.40)* | 0.94 (0.82-1.08) |
| Heart failure | | 1.29 (1.13-1.46)* | 1.63 (1.45-1.84)* |
| Hypertension | | 0.93 (0.87-1.00) | 0.67 (0.59-0.75)* |
| Kidney disease or failure | | 1.18 (1.01-1.38)* | 1.15 (0.99-1.34) |
| Lung cancer | | 1.82 (1.53-2.17)* | 2.96 (2.57-3.40)* |
| Mental disorders | | 1.78 (1.47-2.15)* | 0.71 (0.50-1.00) |
| Myocardial infarction | | 0.98 (0.86-1.13) | 1.08 (0.91-1.28) |
| Obesity | | 1.10 (0.86-1.42) | 0.68 (0.46-1.01) |
| Osteoporosis | | 1.14 (0.99-1.30) | 0.75 (0.62-0.90)* |
| Stroke | | 1.38 (1.20-1.59)* | 1.06 (0.91-1.23) |
| Underweight | | 1.11 (1.00-1.23) | 1.30 (1.17-1.44)* |
|  | | **Need-score home care services** | |
| ADL-score (per 10 points) | |  | 1.12 (1.09-1.15)* |

Table S6. Unadjusted regression results for sociodemographic, –economic factors and comorbidities. CI=Confidence interval. *=significant at 5%-level

| **Outcome:** | | Home care services (n=90,081 observations from 16,738 individuals) | Nursing home or death (n=66,129 observations from 5,291 individuals) |
| --- | --- | --- | --- |
| **Independent variable:** | | Odds ratio (95%-CI) | |
|  | **Sociodemography and -economy** | | |
| Female gender | | 1.17 (1.10-1.24)* | 0.62 (0.57-0.67)* |
| Income per 100,000 NOK | | 0.83 (0.81-0.85)* | 1.00 (0.97-1.03) |
| Education: Secondary vs. primary | | 0.74 (0.69-0.79)* | 1.40 (1.06-1.85)* |
| Education: University/college vs. primary | | 0.55 (0.51-0.60)* | 1.37 (1.02-1.83)* |
| Marital status: Widow/widower vs. married | | 1.65 (1.52-1.80)* | 0.81 (0.72-0.90)* |
| Marital status: Not married/divorced vs. married | | 1.46 (1.37-1.56)* | 0.84 (0.76-0.92)* |
| Permanent disability pension | | 1.81 (1.69-1.94)* | 0.86 (0.76-0.98)* |
|  | **Comorbidities** | | |
| Alcoholism | | 3.17 (2.73-3.67)* | 1.21 (1.00-1.46) |
| Anemia | | 1.98 (1.66-2.36)* | 2.70 (2.40-3.03)* |
| Cancer | | 1.24 (1.14-1.35)* | 2.42 (2.22-2.65)* |
| Cardiovascular disease | | 1.37 (1.28-1.46)* | 1.44 (1.33-1.57)* |
| Dementia | | 3.75 (2.83-4.98)* | 2.48 (2.18-2.83)* |
| Depression | | 2.05 (1.86-2.25)* | 0.89 (0.78-1.02) |
| Diabetes | | 1.32 (1.21-1.44)* | 0.92 (0.82-1.04) |
| Heart failure | | 1.99 (1.77-2.23)* | 2.38 (2.17-2.62)* |
| Hypertension | | 1.02 (0.95-1.08) | 0.73 (0.66-0.81)* |
| Kidney disease or failure | | 1.50 (1.29-1.73)* | 1.73 (1.53-1.96)* |
| Lung cancer | | 2.73 (2.33-3.20)* | 4.26 (3.79-4.79)* |
| Mental disorders | | 2.22 (1.84-2.69)* | 0.61 (0.45-0.84)* |
| Myocardial infarction | | 1.44 (1.27-1.62)* | 1.38 (1.20-1.59)* |
| Obesity | | 1.59 (1.25-2.02)* | 0.68 (0.46-1.01) |
| Osteoporosis | | 1.31 (1.15-1.49)* | 0.99 (0.70-1.39) |
| Stroke | | 1.52 (1.33-1.74)* | 1.25 (1.10-1.43)* |
| Underweight | | 1.39 (1.26-1.54)* | 1.75 (1.58-1.93)* |
|  | | **Need-score home care services** | |
| ADL-score (per 10 points) | |  | 1.20 (1.18-1.22)* |
